# Another Test for Lead Effects: Early Childhood Exposure Influences End-of-Grade Scores

**DOI:** 10.1289/ehp.115-a417b

**Published:** 2007-08

**Authors:** John Tibbetts

Low-level lead exposure has been linked to decreased aptitude—or ability to learn—on standardized IQ tests for school-aged children. Moreover, research studies have suggested that declines in aptitude occur at blood lead levels below the current CDC blood lead action level of 10 μg/dL. Now a team of scientists has studied how lead exposure affects educational achievement—how well children have mastered material taught in school **[*EHP* 115:1242–1247; Miranda et al.]**. The results show that blood lead levels far lower than 10 μg/dL in early childhood correlate with lower educational achievement in elementary school as measured by performance on end-of-grade (EOG) tests.

Data for the study came from two large databases generated by two different offices of the State of North Carolina for the same population but at different time periods. Blood surveillance data were provided by a state registry for seven adjacent North Carolina counties. The scientists used screening data from 1995 through 1998 for 35,815 children. For children who were screened more than once, the researchers used the highest blood lead level recorded. During this period, an estimated 21.9–30.4% of North Carolina children aged 1 and 2 years were screened for lead.

The North Carolina Education Research Data Center provided educational testing data from 2000–2004 for fourth-grade students in the seven-county study region. In North Carolina, each child in grades 3 through 8 takes a multiple-choice EOG test in reading and mathematics.

The researchers linked the two separate data sets to locate records of children who had been screened for lead and had also taken at least one EOG test. To ensure accuracy, the researchers used 16 different combinations of identifiers, including Social Security numbers, date of birth, the county’s Federal Information Processing Standards code, and first and last name. This process linked 42.2% of screened children to at least one EOG record.

The scientists found a strong dose–response effect between early childhood lead exposure and performance on elementary school achievement tests. Childhood blood lead levels as low as 2 μg/dL at age 1 or 2 years had a discernible correlation with deficits in later EOG testing. A blood lead level of 4 μg/dL was associated with a significant decline in EOG reading and math scores, with an impact nearly equal to that of participating in the free or reduced lunch program, the classic poverty indicator in school data. The researchers want to follow the same children through their elementary, middle school, and high school years to assess the persistence of the effects found in this study.

## Figures and Tables

**Figure:**
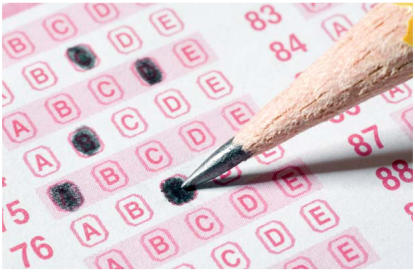
Another angle on lead Lead exposure has long been known to affect aptitude. New results show achievement also is at risk, and at levels below CDC recommendations.

